# Pharmacokinetics of ponazuril after administration of a single oral dose to green turtles (*Chelonia mydas*)

**DOI:** 10.1080/01652176.2021.2008045

**Published:** 2021-12-08

**Authors:** Elliott R. Jacobson, Nicole I. Stacy, Douglas R. Mader, Richie Moretti, Bette Zirkelbach, Olivia Carlile, Courtney D. O’Connor, Kaylin J. Caperton, Lara K. Maxwell

**Affiliations:** a Department of Small Animal Clinical Sciences, College of Veterinary Medicine, University of Florida, Gainesville, FL, USA; b Department of Comparative, Diagnostic, and Population Medicine, University of Florida, Gainesville, FL, USA; c The Turtle Hospital, Marathon, FL, USA; d Department of Physiological Sciences, College of Veterinary Medicine, Oklahoma State University, Stillwater, OK, USA

**Keywords:** Sea turtle, *Chelonia mydas*, triazine, antiparasitic, *Caryospora*, coccidia

## Abstract

The coccidian protozoan, *Caryospora cheloniae*, has been associated with severe enteritis and encephalitis in immature farm-raised green turtles (*Chelonia mydas*) in the Cayman Islands, immature green turtles off the coast of Florida, and immature stranded sea turtles in Australia. An effective anti-coccidial drug that is both orally absorbed and well-distributed throughout the body is needed for treatment of turtles diagnosed with coccidiosis in rehabilitation facilities. Ponazuril is a triazine antiprotozoal drug that is approved in the USA for the treatment of another Apicomplexan, *Sarcocystis neurona,* and has also been successfully used in the therapy of other coccidian parasites. The objective of this study was to perform an oral dose-ranging pilot study (10–100 mg/kg of body weight ponazuril) in green turtles (N = 9), followed by oral administration of ponazuril at 100 mg/kg body weight (N = 8) to assess its disposition. Another goal of this study was to optimize the method of oral drug administration to green turtles. Plasma ponazuril concentrations were quantified using high performance liquid chromatography (HPLC). Standard compartmental models were fit to the data. Ponazuril was absorbed after oral administration at 100 mg/kg BW, with a maximum plasma concentration of 3.3 µg/ml. Dose-dependent pharmacokinetic parameters only weakly correlated with the dose rate, apparently due to considerable pharmacokinetic variability observed between turtles. Administration of ponazuril in gelatin capsules using a balling gun was deemed the least variable and most successful method of drug administration. Further studies are needed to evaluate the safety and efficacy of ponazuril in sea turtles with coccidiosis.

## 1. Introduction

The phylum Apicomplexa is a large and diverse group of protozoan parasites, many of which are the causative agents of intestinal and extra-intestinal lesions in both domestic and wild animals (Morrison 2009). The apicomplexan coccidia, *Caryospora cheloniae*, was first identified as a pathogen in farmed 4–8-wk-old green turtles (*Chelonia mydas*) in the Cayman Islands, British West Indies (Leibovitz et al. 1978) and in immature green turtles stranded on beaches in Australia (Gordon et al. 1993). More recently, several outbreaks of caryosporosis have been reported in immature stranded green turtles in Florida, USA (Stacy et al. 2019). Caryosporosis is associated with encephalitis, which is usually lethal (Gordon et al. 1993; de Gouvea Pedroso et al. 2020). Of 63 green turtles that were stranded in Australia and diagnosed with coccidiosis, 54 (86%) were euthanatized or died (de Gouvea Pedroso et al. 2020).

Ponazuril is a triazine anticoccidial drug that targets the apicoplast and tubular mitochondrion of the organism and has been used to treat coccidian infections in a wide variety of animals (Darius et al. 2004). In limited reptile studies, anticoccidial effects were reported when ponazuril was orally administered to bearded dragons (*Pogona vitticeps*), red-footed tortoises (*Chelonoidis carbonaria*) and green turtles (Bogoslavsky 2007; Pelton et al. 2013; Benge et al. 2018). Although ponazuril has been used to treat green turtles with *C. chelonei* infection, the optimal dosage and pharmacokinetic parameters in sea turtles are unknown to date. Because of this need for clinical management of sea turtles with caryosporosis, we designed a ponazuril pharmacokinetic study in order to determine an appropriate dosing schedule in green turtles. We also investigated several methods of oral drug administration in order to optimize the dosing methodology in this species.

## 2. Materials and methods

### 2.1. Ethics statement

All samples were collected under Florida Fish and Wildlife Commission Marine Turtle Permits 19-021 and 20-021. This project was approved by the University of Florida Institutional Animal Use and Care Committee, protocol #201706823.

### 2.2. Study animals

Seventeen unsexed immature green turtles at the Turtle Hospital in Marathon, Florida Keys, USA, were utilized in this study. These animals had previously stranded in the Florida Keys and along the coast of Florida. Sea turtles at the Turtle Hospital are treated for fibropapillomatosis, boat strike/trauma, emaciation, cold stunning, and line entanglement. The study turtles (n = 17) had a mean weight of 17.0 ± 7.6 (S.D.) kg (ranging in weight from 4.5 kg to 29.9 kg) and had a mean straight carapace length (SCL) of 49.5 ± 7.8 cm (ranging in length from 31.3 to 64.5 cm), and were in good physical condition based on physical examination, feeding behavior, blood analysis for hematology and plasma biochemistry, and were considered either successfully rehabilitated or clinically normal permanent captives (i.e. no longer releasable due to permanent injuries). Study turtles did not receive any other medications during the study period. The green turtles were fed two times daily with a diet consisting of romaine lettuce, green peppers and floating catfish chow (Purina Catfish Chow, Purina Mills, LLC, St. Louis, MO, USA). The diet was supplemented with Mazuri Sea Turtle Supplement (from 0.25 to 1.5 g daily, depending on size of turtle; Mazuri Exotic Animal Nutrition, St. Louis, MO, USA) and additional calcium (150–600 g daily, depending on size of turtle; Nature Made, West Hills, CA, USA). The supplements were fed to the turtles in squid. The turtles were housed outdoors in 9.1 m diameter, 1.5 m high circular custom-made fiberglass tanks (100,080 l) or in 1.8 m diameter, 76 cm high fiberglass tanks (2002 l) in salt water ranging in temperature between 24 and 30 °C. All turtles were exposed to a natural light/dark cycle.

### 2.3. Experimental design

#### 2.3.1. Phase I, pilot study

Nine green turtles were used in the pilot study. Turtles ranged in weight from 4.5 to 29.25 kg (12.5 ± 7.2, mean ± SD). An initial blood sample (2 ml) was collected prior to oral administration of ponazuril (Marquis, Bayer Corp., Shawnee Mission, Kansas, USA). Each turtle received one dose of 10 (N = 2), 20 (N = 2), 30 (N = 1), 50 (N = 2) or 100 (N = 2) mg/kg body weight orally. Oral administration was accomplished either by tubing a turtle the calculated amount (N = 2) or administering the calculated amount in porcine hard gelatin capsules (Torpac Inc., Fairfield, NJ, USA) that were placed in squid and fed to turtles (N = 7). Blood samples (2 ml) were obtained from the dorsal cervical sinus or supervertebral vein, collected in 3 ml syringes attached to a 2.54 cm, 22 g needle (Coviden Monoject, Thermo Fisher Scientific, Waltham, MA, USA) that had been coated with lithium heparin followed by expulsion of all visible liquid, then transferred to 3 ml blood collection tubes (Vacutainer, Becton Dickinson and Company, Franklin Lakes, USA) containing lithium heparin. Blood samples were collected from each turtle at 1, 6, 12, 24, 48, 72, 96 h, and 7, 14, 21 and 30 days after ponazuril administration. Tubes were placed on ice and centrifuged within 15 minutes after sample collection. Following centrifugation, plasma was transferred into 2 ml cryotubes (Corning Inc, Corning, NY, USA), and frozen at −80 °C until analysis.

#### 2.3.2. Phase II study

Based on the data collected in the Phase I, pilot study, eight turtles were administered a single oral dose of 100 mg/kg body weight ponazuril. Turtles ranged in weight from 15.6 to 29.9 kg (22.0 ± 4.9 kg). All turtles in the Phase II study received ponazuril in a gelatin capsule that was orally administered using a commercially obtained balling gun (Neogen, Lansing, MI, USA).

#### 2.3.3. Ponazuril assay

Plasma ponazuril concentrations were quantified using high performance liquid chromatography (HPLC). Ponazuril and toltrazuril were purchased from Sigma-Aldrich Corp. (St. Louis, MO, USA), while reagents such as formic acid, acetonitrile, and methanol from Fisher Scientific (Waltham, MA, USA), and columns from Waters Corp (Milford, MA, USA). Since large endogenous peaks co-eluted with ponazuril when simple protein precipitation plasma clean-up methods were employed, solid phase extraction (SPE) was used for sample clean-up before analysis. The final method was simple, sensitive and specific for quantitation of ponazuril in green turtle plasma.

For each experimental, quality control, or calibration sample, 500 µL of plasma was mixed with 10 µL of the internal standard, toltrazuril, prepared at a concentration of 10 µg/ml in methanol. Each sample was then mixed with an equal volume of 2% formic acid before being loaded onto conditioned 1 cc Oasis HLB SPE cartridges. The cartridges were washed with 1 ml of 5% methanol, and samples were eluted with 1 mL of methanol, then dried under nitrogen at 40 °C. Samples were resuspended in 150 µl of mobile phase before 25 µL were injected onto the HPLC system (Waters Corporation, Milford, MA, USA). The HPLC consisted of a 1500 series binary pump, 717 autosampler, column heater at 35 °C, and a 4.6 × 250 mm, 5 µm, endcapped RP18 column. Analytes were eluted isocratically using 0.1% formic acid in 38% water, 31% methanol, and 31% acetonitrile. Ultraviolet absorbance was detected at 255 nm.

The calibration curve was constructed from the ratio of the peak height of ponazuril:toltrazuril versus the nominal concentration, with a 1/concentration weighting scheme. Calibration curves were constructed using plasma from unmedicated sea turtles that were fortified with 25 µL of methanol containing appropriate concentrations of ponazuril (Sigma-Aldrich Corp.). Final concentrations of ponazuril in plasma were 0.05, 0.125, 0.25, 0.5, 1, 2.5, 5, 10, 25 and 50 µg/ml. Intraday accuracies at concentrations of 0.5 µg/ml and 25 µg/ml were 109% and 108%, whereas coefficients of variation (CV) were 8% and 7%, respectively. Interday accuracies at concentrations of 0.5 µg/ml and 25 µg/ml were 102% and 114%, whereas CVs were 11% and 6%, respectively. The limit of quantitation (LOQ) was determined to be 0.05 µg/ml and was defined as the lowest concentration at which the accuracy was within 20% of the nominal concentration and the CV less than 20%. The limit of detection was not determined.

#### 2.3.4. Pharmacokinetic analysis

##### 2.3.4.1. Phase I study

Plasma ponazuril concentrations from the pilot study were analyzed compartmentally by nonlinear regressionusing Kinetica™ software version 5.0 (Thermo Fisher Scientific). Data from each turtle were fit by the following equation:

$$C=\sum_{i=1}^{n}A_{i}\cdot e^{-\alpha t}$$

Standard compartmental models with first order absorption were fit to the data, and the most appropriate model was selected using Aikaike’s information criterion (Yamaoka et al. 1978). Standard compartmental pharmacokinetic equations were used to estimate the pharmacokinetic parameters reported for each turtle. Since a range of ponazuril dose rates were administered during the pilot study, the relationships between oral dose rate and AUC, T_lag_, and t_1/2(Ke)_ were assessed by simple linear regression using Excel (Microsoft Corp.).

##### 2.3.4.2. Phase II study

The pilot study data were used to inform the design of the Phase II, single dose study. Turtles were visually observed for evidence that all capsules were swallowed after ponazuril administration and were categorized as being successfully or unsuccessfully dosed. Pharmacokinetic analysis of the Phase II study data was performed compartmentally, as described above. Superposition was then applied to the individual data from each turtle in the successful administration group, to predict the plasma ponazuril concentrations expected from the administration of multiple doses of ponazuril (Thron 1974). The mean predicted plasma ponazuril concentrations were constructed for the proposed multiple dose regimen.

## 3. Results

### 3.1. Phase I study

**Figure 1.**
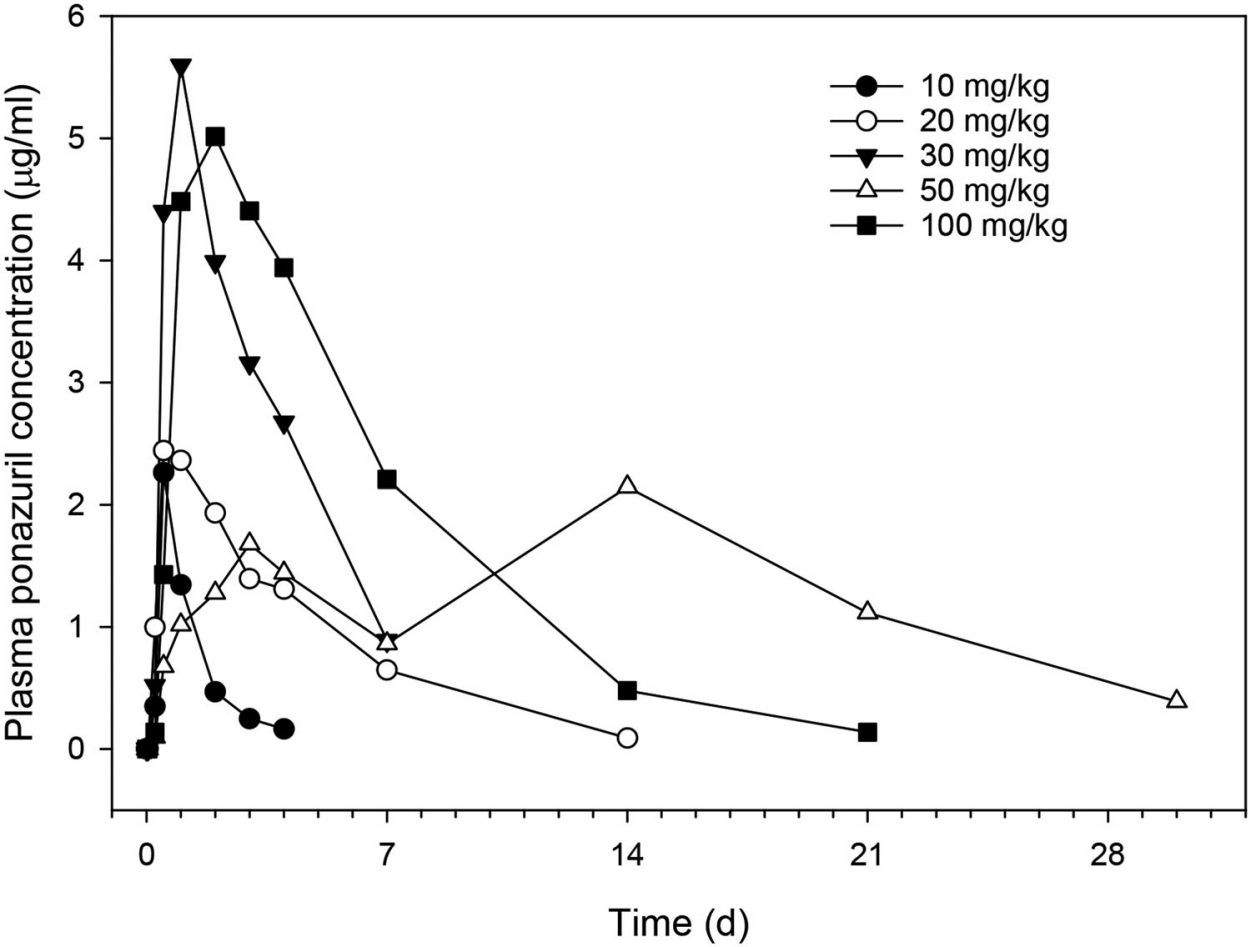
Plasma ponazuril concentration versus time curves after oral administration of ponazuril to green turtles (*Chelonia mydas*) in the dose escalation pilot study. Each data point is a mean of two turtles, except the 30 mg/kg BW dose rate for which only one turtle was studied.

Plasma ponazuril concentrations reached maximal values between one and three days after oral administration to most turtles and could be quantified for up to 30 d after administration of a single dose, in a dose-rate dependent manner (Figure 1). A one-compartment model with first-order absorption adequately described the plasma ponazuril versus concentration data. However, the use of a lag time until first-order absorption occurred was necessary to adequately fit the data. The pilot study used two different administration techniques in an attempt to produce consistent ponazuril disposition. Administration by tubing produced particularly low plasma ponazuril concentrations in the two turtles subject to this method of administration (Table 1), so this method was replaced with ponazuril filled capsules inserted into food. For either method of administration, absorption of ponazuril was slow and highly variable, with a mean T_max_ of 56 hr and range of 12–264 hr (Table 1). The apparent elimination half-life was also prolonged and variable, with a mean of 94 hr and range of 23–256 hr. Other pharmacokinetic parameters were similarly variable.

**Table 1.** Pharmacokinetic parameters after administration of various oral dose rates of ponazuril to nine green turtles (*Chelonia mydas*) in the pilot study.

Admin	Dose	C_max_/	AUC/	K_abs_	T_lag_	T_max_	t_1/2(Ke)_	MRT	Vz/F
Method	mg/kg BW	Dose	Dose	h^-1^	h	h	h	h	l/kg
Tube	10	0.03	3	0.09	2.0	28	59	84	28
Squid	10	0.25	9	5.92	11	12	23	23	4
Tube	20	0.03	3	0.06	6.0	35	36	46	19
Squid	20	0.23	26	0.29	4.3	16	70	96	4
Squid	30	0.18	18	0.18	5.6	21	56	76	4
Squid	50	0.04	29	0.01	6.0	264	256	364	13
Squid	50	0.03	5	0.15	5.2	26	85	117	27
Squid	100	0.1	14	0.08	9.0	41	76	101	8
Squid	100	0.01	2	0.05	6.0	61	184	260	110
	Mean	0.10	12	0.76	6.1	56	94	130	24
	SD	0.10	10	1.94	2.5	79	76	110	34

Admin method = paste was administered by tube or as ponazuril filled capsules inserted into squid, Dose = dose rate, C_max_ = maximal calculated plasma drug concentration normalized to dose rate, AUC_∞_ = area under the plasma drug concentration-time curve, extrapolated to infinity and normalized to dose rate, Kabs = absorption rate, Tlag = lag time of absorption, T_max_ = calculated time of maximal measured plasma drug concentration, t_1/2(Ke)_ = apparent elimination phase half-life, V_z_/F = apparent volume of distribution divided by bioavailability.

**Figure 2.**
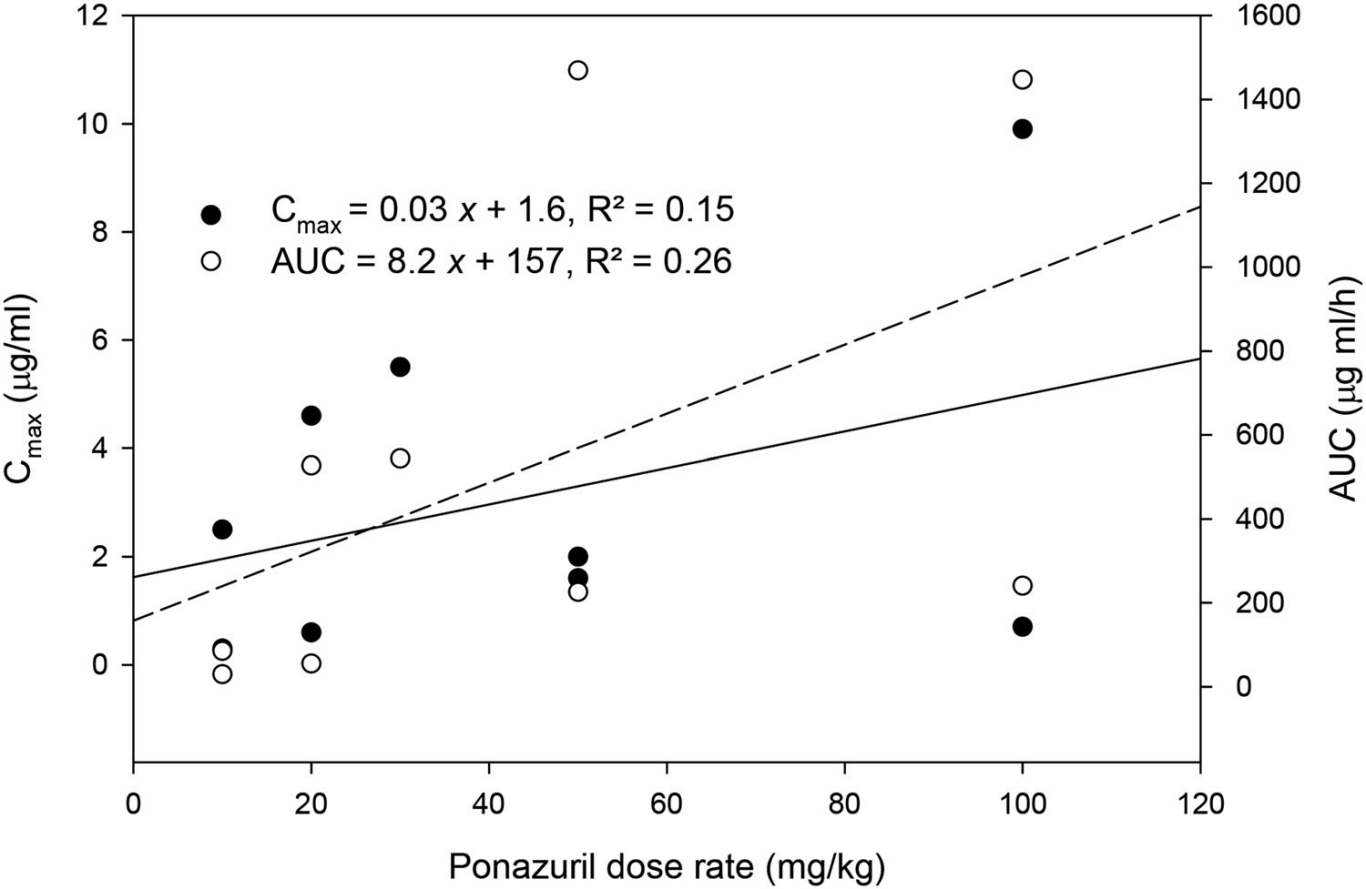
Relationship between AUC or C_max_ versus the oral dose rate of ponazuril in the pilot study.

**Figure 3.**
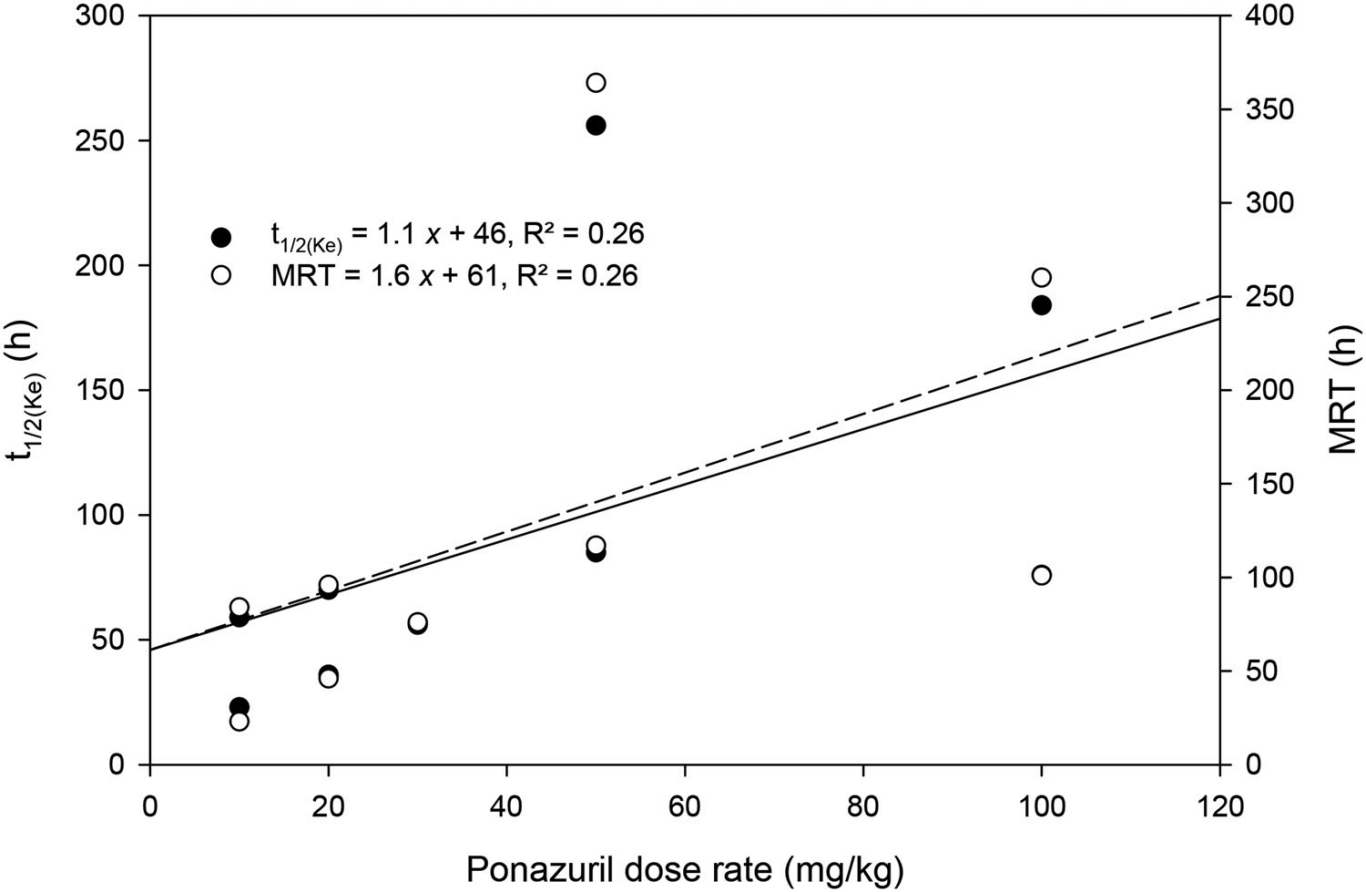
Relationship between t_1/2(Ke)_ and MRT versus the oral dose rate of ponazuril administration to green turtles (*Chelonia mydas*) in the pilot study.

**Figure 4.**
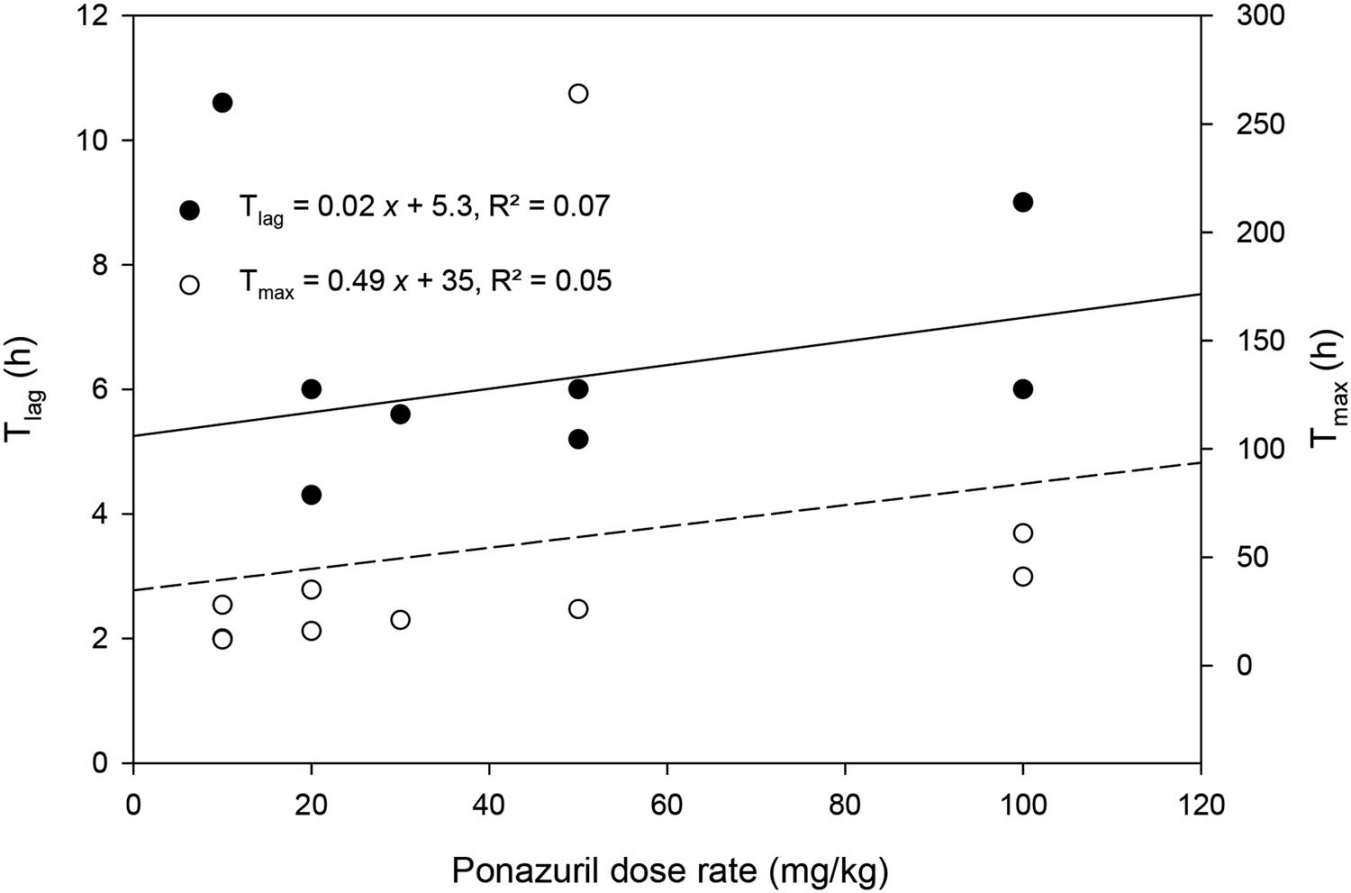
Relationship between T_lag_ and T_max_ versus the oral dose rate of ponazuril administration to green turtles (*Chelonia mydas*) in the pilot study.

There was only a weak relationship between peak plasma ponazuril concentration (C_max_) or area under the plasma concentration versus time curve (AUC) and dose rate (Figure 2). Several other parameters that are independent of dose if first-order kinetics are observed, such as t_1/2(Ke)_ and MRT, were more strongly related to dose rate (Figure 3). Absorption related parameters did not vary with dose rate (Figure 4).

### 3.2. Phase II study

**Figure 5.**
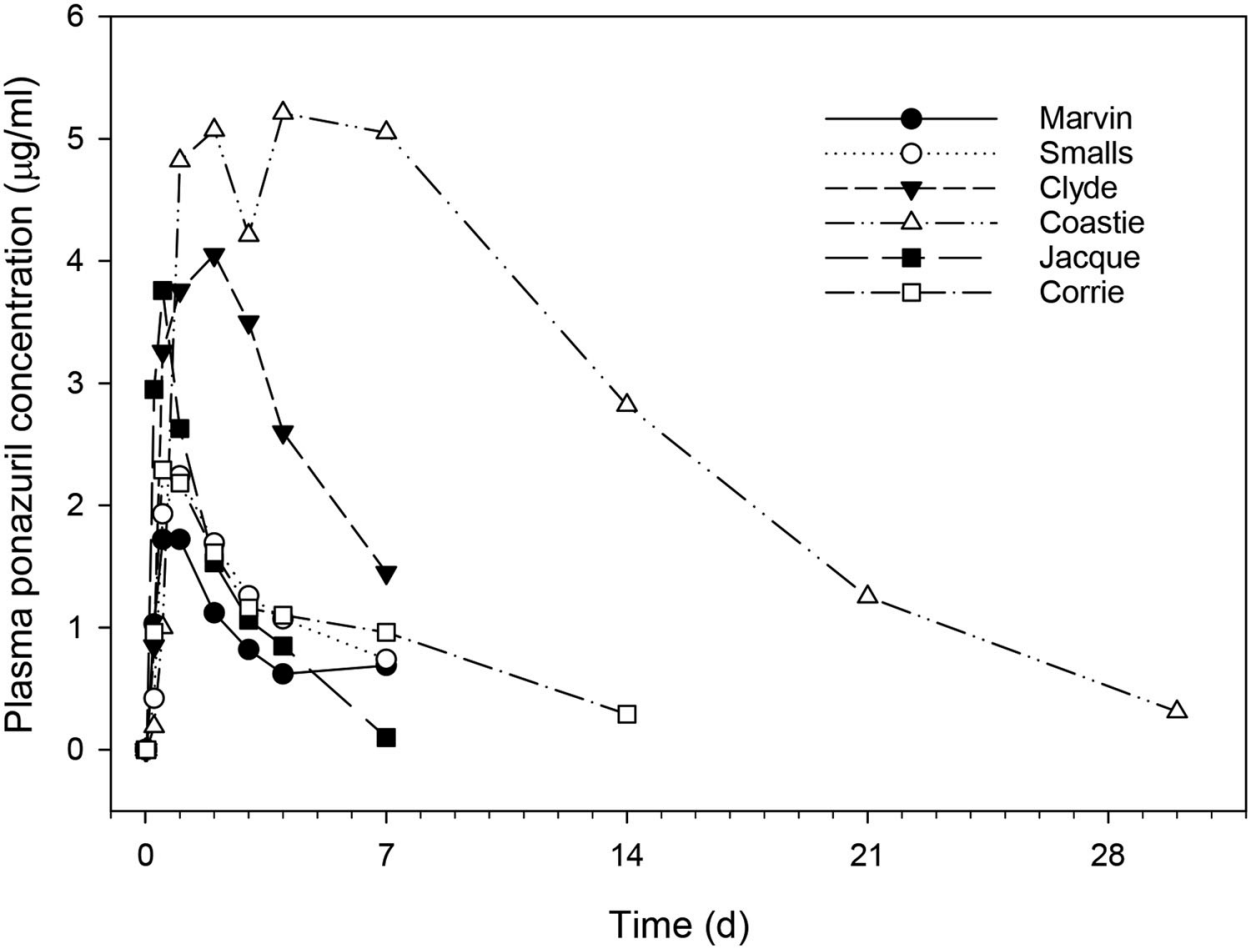
Plasma ponazuril concentration versus time curves for six green turtles successfully administered a single oral dose of ponazuril-filled capsules at a dose rate of 100 mg/kg BW.

Turtles were observed after administration of ponazuril-filled capsules by balling gun. Administration appeared to be successful in six of eight turtles. However, in two turtles, ponazuril paste was visibly expelled from the mouth and nares when the turtle entered water, soon after administration. For the two turtles in which problematic administration was identified, both turtles had lower C_max_ and AUC values as compared with the successfully dosed turtles (Table 2). Therefore, data from turtles in Phase II were separated into those where successful versus problematic administrations of ponazuril were observed. Within the group with successful administration, plasma ponazuril concentration versus time curves appeared to be less variable among most turtles as compared with the pilot study data, although the curve associated with one turtle (Coastie) was substantially greater than that of the other turtles (Figure 5). The data from that single turtle were responsible for most of the variation seen between successfully dosed turtles in this phase of the study. The C_max_ was fairly consistent between turtles, with a mean of 3.3 µg/ml and range of 1.7–5.6 µg/ml. A lag time was again necessary to adequately fit the one-compartment model with first-order absorption to the data, and the T_lag_ was similar to that observed in the pilot study.

**Table 2.** Pharmacokinetic parameters for ponazuril following a successful single oral dose of 100 mg/kg BW to six green turtles (*Chelonia mydas*) or problematic administration to two green turtles.

Pharmacokinetic parameters	Successful dose N = 6	Problematic dose N = 2
C_max_ (µg/ml)	3.3 ± 1.5	0.9 ± 0.5
T_max_ (h)	22 ± 14	18 ± 3
AUC_∞_ (µg·h/ml)	528 ± 634	74 ± 39
K_abs_ (h^−1^)	2.4 ± 4.2	0.5 ± 0.2
t_1/2(abs)_ (h)	0.29* ± 1.7	1.7 ± 0.8
T_lag_ (h)	8.4 ± 7.5	9.6 ± 0
K_e_ (h^−1^)	0.011 ± 0.006	0.014 ± 0.001
t_1/2(Ke)_ (h)	60* ± 34	50 ± 4.2
V_z_/F (l/kg)	33 ± 17	114 ± 68

Values are expressed as the mean or *harmonic mean ± s.d. C_max_ = maximal calculated plasma drug concentration, T_max_ = calculated time of maximal measured plasma drug concentration, AUC_∞_ = area under the plasma drug concentration-time curve, extrapolated to infinity, K_abs_ = absorption rate, t_1/2(abs)_ = absorption rate half-life, T_lag_ = lag time of absorption, K_e_ = apparent elimination rate, t_1/2(Ke)_ = apparent elimination phase half-life, V_z_/F = apparent volume of distribution divided by bioavailability.

**Figure 6.**
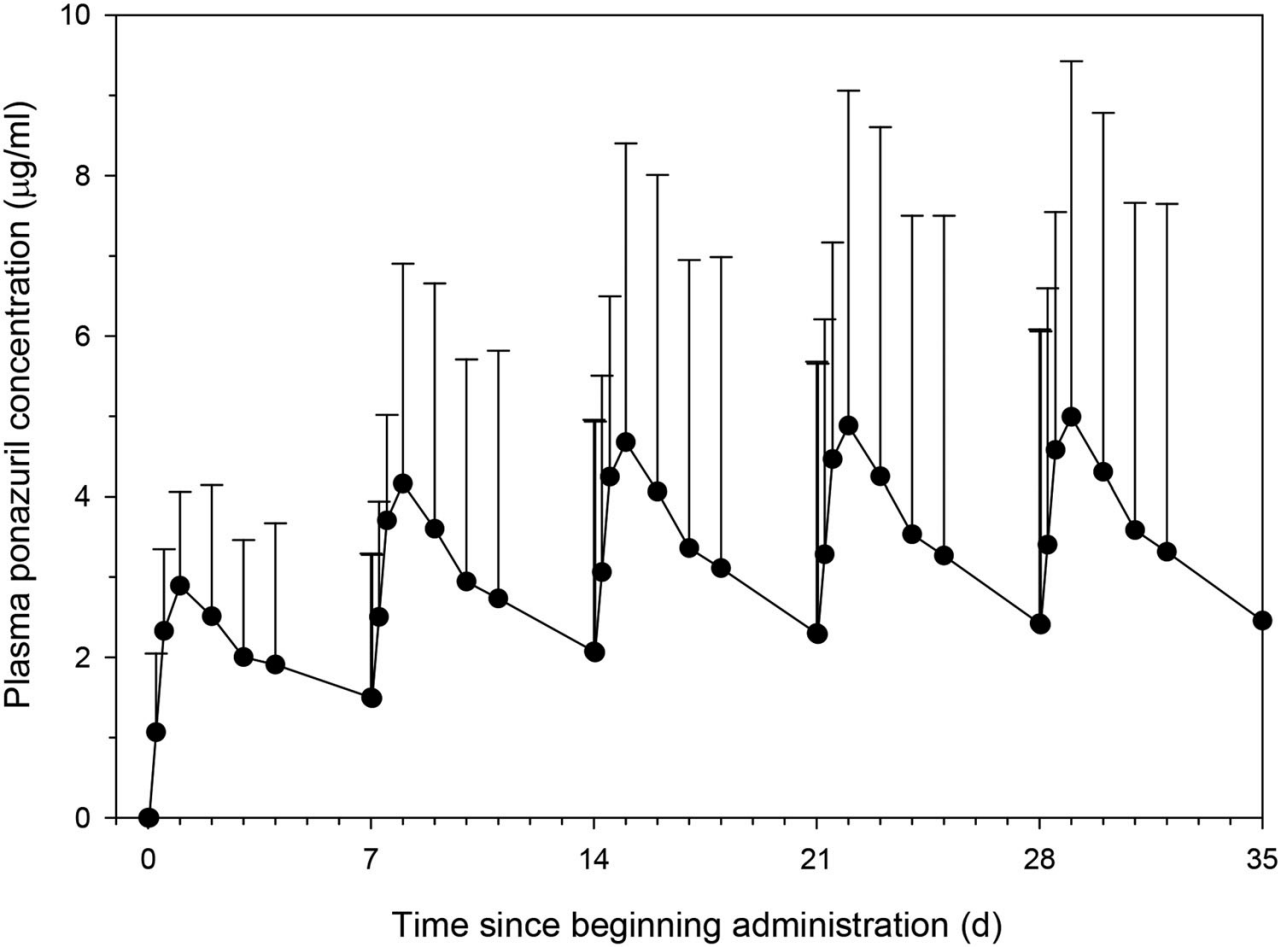
Predicted (mean ± SD) plasma ponazuril concentrations after oral administration of ponazuril at a dose rate of 100 mg/kg BW to green turtles (*Chelonia mydas*) every 7 days.

Plasma ponazuril concentrations resulting from multiple dose administrations were predicted from the single dose data in each of the successfully dosed turtles using a dosing regimen of 100 mg/kg and a dosing interval of one week. Ponazuril accumulation was predicted to occur with this dosing regimen, with approximately steady state conditions reached about two weeks after beginning drug administration (Figure 6; Table 3). This dosing regimen was predicted to result in mean peak concentrations of 4.9 µg/ml by the fourth dose, with trough concentrations of 2.4 µg/ml. However, the predicted peaks and troughs were associated with considerable inter-individual variability. In 3/6 turtles for which multiple dose data were predicted, trough plasma ponazuril concentrations were predicted to exceed 1 µg/ml after the fourth weekly dose, with one outlier turtle (Coastie) predicted to have a fourth dose trough concentration of 10 µg/ml. If this outlier turtle was removed from the analysis, then the mean ± SD trough after the fourth dose would be 0.9 ± 0.6 µg/ml, and approximately steady state conditions would be reached after the second dose.

**Table 3.** Mean ± SD plasma ponazuril concentrations of peaks (C_max_) and troughs (C_min_) predicted from single dose data in six green turtles (*Chelonia mydas*) if 100 mg/kg BW oral ponazuril were administered every 7 days for four doses.

	Peak (µg/ml)	Trough (µg/ml)
Dose 1	2.9 ± 1.2	1.5 ± 1.8
Dose 2	4.2 ± 2.7	2.1 ± 2.9
Dose 3	4.7 ± 3.7	2.3 ± 3.4
Dose 4	4.9 ± 4.2	2.4 ± 3.7

## 4. Discussion

This study reports pharmacokinetic parameters of ponazuril after oral administration to green turtles. Oral administration via gelatin capsules and balling gun reached plasma concentrations of ponazuril that are considered therapeutic for treatment of coccidial infections in mammals. This information will be useful for the development of further pharmacokinetic studies and optimized treatment protocols for sea turtles affected by *Caryospora cheloniae*.

Three different methods of oral ponazuril administration were investigated in the current study. Oral tubing of ponazuril produced surprisingly low plasma ponazuril concentrations, so this method was discontinued after use in some turtles during the pilot study. It may be that the presence of keratinized papillae, diverticula, and an S-shaped bend in the esophagus of green turtles may have hindered successful tube placement or ponazuril absorption by this route (Magalhães et al. 2012). Administration of ponazuril-filled capsules inserted into squid was the least stressful technique to the turtles, as well as the simplest method of oral administration since restraint was not required. However, green turtles unpredictably crushed the capsules open during squid consumption, which may account for the high inter-individual variability observed with this method of administration. This variability might also explain the weak correlation between pharmacokinetic parameters that are dose-dependent if first-order kinetics are observed (C_max_ and AUC) and the ponazuril dose rate. Another possible explanation for the weak correlation between C_max_ and AUC with the dose rate is that nonlinear disposition processes, such as saturable absorption or elimination, occurred as the dose rate increased. However, since T_lag_ and T_max_ did not depend on the dose rate, saturable absorption kinetics were unlikely. Several parameters that can be related to saturable elimination, t_1/2(Ke)_ and MRT, did weakly depend upon dose rate. However, inspection of the data revealed that the most likely explanation for this relationship was that the lower dose rates were associated with such low plasma ponazuril concentrations that they could only be quantified for a short period of time. In contrast, the higher ponazuril dose rates produced higher ponazuril concentrations that could be quantified for much longer. Given that both the quantifiable period and the dose rate varied together, it was difficult to separately evaluate whether a longer detection period or saturable elimination was responsible for this weak relationship.

For the Phase II study, administration of ponazuril-filled capsules using an oral balling gun appeared to work well for six of eight turtles. However, given that ponazuril administration was also problematic in two of the turtles, oral administration of ponazuril continued to be associated with considerable inter-individual variability, even when administered as capsules by balling gun. Since multiple doses of ponazuril were not administered, we were unable to separately assess whether intra-individual variability would also impact ponazuril disposition. However, the large variability in ponazuril disposition observed in green turtles was similar to that reported when ponazuril was administered orally at a dose rate of 20 mg kg^−1^ to red-footed tortoises, *Chelonoidis carbonaria* (Benge et al. 2018). The plasma ponazuril concentrations of both chelonian species were lower than expected as compared with disposition in mammalian species. The rate of oral absorption of ponazuril in *C. carbonaria* was exceedingly slow, with a mean T_max_ of 144 h, as compared with the green turtles in Phase II, where the mean T_max_ was 22 h. The lag time that was necessary to fit the plasma concentration versus time data in green turtles was presumably due to the time necessary for capsules to be digested, releasing the ponazuril. Lag time might also have represented transit time to an absorptive area of the digestive tract, since even the two turtles that were administered ponazuril via tubing required a lag time to fit the data. Since caryosporosis is associated with enteritis in green turtles, affected turtles might absorb ponazuril differently than the healthy turtles employed in the current study. Gastrointestinal disease can impact drug absorption as a consequence of pathophysiological changes (Effinger et al. 2019). For example, if gut transit is slowed, then the T_max_ would be longer and the C_max_ lower, though the AUC and trough concentrations could be relatively unchanged. Other aspects of enteral disease, such as denuding of villi, can decrease total drug absorption, leading to poor drug efficacy. Loss of gut mucosal integrity could increase drug absorption, possibly leading to toxicity. Further study of affected turtles would better define the best therapeutic regimen.

The terminal phase half-life was quite variable, especially in the pilot study, where the administration of different dose rates resulted in various quantifiable periods of plasma ponazuril. In the Phase II study, the mean terminal phase half-life was 60 h, shorter than the approximately 4 d elimination half-life reported in horses. In the green turtles of the current study, the terminal phase half-life could not be unambiguously assigned as the elimination half-life because an intravenous route of administration was not compared with the oral route of administration. This lack of discrimination allows for the possibility that flip-flop kinetics occurred, and that the terminal phase actually represents ponazuril absorption. Nonetheless, it is likely that the terminal phase truly represented the elimination of ponazuril in the green turtles, as it was much slower than the apparent absorption rate and was more consistent with previous reports of the slow elimination rate of ponazuril in other veterinary species (Furr and Kennedy 2001).

Superposition was used to predict the plasma ponazuril concentrations that would result if 100 mg/kg BW ponazuril was administered to green turtles as capsules by balling gun in Phase II. The maximum studied dose, 100 mg/kg BW, was chosen for superposition with a dosing interval of 7 days, which was deemed a practical interval for an administration technique that requires restraint. Most turtles were predicted to reach similar plasma ponazuril concentrations, but one turtle was predicted to have far higher concentrations than the others, with predicted peak and trough concentrations of 15.3 and 12.4 µg/ml, respectively, after the fourth weekly dose. Since the predicted plasma ponazuril concentrations were subject to considerable interindividual variability, safety and response to treatment might be similarly variable. However, predicted steady state plasma ponazuril concentrations in green turtles with the proposed dosing regimen were not very different from the ponazuril concentrations that have been reported in mammalian species that are regularly treated with this drug.

To our knowledge, plasma ponazuril concentrations necessary to inhibit *Caryospora cheloniae* or other reptile protozoan parasites have not been reported to date. However, *in vitro* inhibition of ponazuril against other apicomplexan parasites has been reported, such as the reported ponazuril IC_50_ of the coccidian parasite *Cystoisospora suis* of 3.4 µg/ml (Joachim and Ruttkowski 2021). Several studies have also described inhibition of *Sarcocystis neurona* using *in vitro* systems, though a limited range in ponazuril concentrations have been tested (Lindsay et al. 2000; Mitchell et al. 2005). The lowest tested ponazuril concentration of 5 µg/ml inhibited *S. neurona* in one study, but another study that included a range of concentrations found that the IC_50_ would be <0.1 µg/ml.

This inhibitory concentration of less than 1 µg/ml is consistent with the low ponazuril cerebrospinal fluid (CSF) concentrations reported in horses, the veterinary species for which ponazuril use is approved for the therapy of equine protozoal myeloencephalitis (EPM) caused by *S. neurona*. In horses, ponazuril is approved for daily oral administration of 5 mg/kg BW, and this dosing regimen produces mean serum concentrations of 5.33 µg/ml with accompanying CSF concentrations of 0.162 µg/ml (Furr and Kennedy 2001). Ponazuril CSF concentrations were not assessed in the current study of green turtles, but the mean predicted maximal plasma ponazuril concentration of 3.3 µg/ml after the first dose was similar to equine steady state serum concentrations. Modeling predictions of the current green turtle data suggested that if a dose rate of 100 mg/kg BW ponazuril was administered orally every seven days, then there would be approximately 50–70% fluctuation in plasma ponazuril concentrations over the dosing interval. Mean peak and trough plasma ponazuril concentrations in green turtles would also be similar to that reported in horses, albeit more variable in the turtles. In the absence of *in vitro* inhibitory data for *C. cheloniae,* these comparisons between equine and green turtle ponazuril disposition indicate that the proposed dosing regimen, 100 mg/kg BW ponazuril in capsules administered every seven days, warrants further study in green turtles. Further assessment of orally administered ponazuril disposition in green turtles affected with caryosporosis would also serve to refine and optimize the proposed therapeutic regimen of this promising antiprotozoal drug in the presence of enteritis.
